# Alterations in short-chain fatty acids and serotonin in irritable bowel syndrome: a systematic review and meta-analysis

**DOI:** 10.1186/s12876-020-01577-5

**Published:** 2021-01-06

**Authors:** Mei Luo, Xiaojun Zhuang, Zhenyi Tian, Lishou Xiong

**Affiliations:** Department of Gastroenterology and Hepatology, the First Affiliated Hospital of Sun Yat-Sen University, Guangzhou, China

**Keywords:** Irritable bowel syndrome, Short-chain fatty acids, Serotonin, Brain-gut-microbiota axis, Meta-analysis

## Abstract

**Background:**

Short-chain fatty acids (SCFAs) and serotonin (5-hydroxytryptamine, 5-HT) may be associated with the pathogenesis of irritable bowel syndrome (IBS). There are some reports of alterations in SCFAs and 5-HT in IBS, but their results are inconsistent. We aimed to perform a meta-analysis to assess alterations in SCFAs and 5-HT in IBS patients and their potential role in the abnormal brain-gut-microbiota (BGM) axis.

**Methods:**

Case–control studies detecting SCFAs and 5-HT in IBS patients were identified from PubMed, Web of Science, Cochrane Library, and Scopus databases to identify relevant articles up to September 2018. The standardized mean differences (SMDs) with 95% confidence intervals (CIs) of SCFAs and 5-HT were calculated by REVIEW MANAGER 5.3 to evaluate the alterations of 5-HT and SCFAs in IBS.

**Results:**

Five studies on SCFAs and 5 on 5-HT in IBS patients were included. As compared to healthy controls (HCs), the SMDs of 5-HT in IBS patients was 2.35 (95% CI 0.46–4.24) and the SMDs of total SCFAs, acetic acid, propionic acid, and butyric acid in IBS patients were − 0.01 (95% CI − 0.57–0.55), − 0.04 (95% CI − 0.55–0.47), 0.07 (95% CI − 0.45–0.60), and − 0.00 (95% CI − 0.49–0.49), respectively.

**Conclusions:**

There was an increase in 5-HT in blood of IBS patients, indicating the increased 5-HT in blood may be involved in IBS pathogenesis. However, there were no significant differences in SCFAs in feces between IBS patients and HCs. But the study did not differentiate between subgroups of IBS. These findings might provide insight for future studies of the BGM axis in the pathogenesis of IBS.

Mei Luo and Xiaojun Zhuang contributed equally to the writing of this article

## Background

Irritable bowel syndrome (IBS) is one of the most common functional gastrointestinal disorders with uncertain pathogenesis, and serious clinical symptoms (such as abdominal pain, diarrhoea or constipation) will have a significant impact on the quality of life in IBS patients [1, 2]. Visceral hypersensitivity, altered gastrointestinal permeability, immune dysfunction or psychological disorder have been generally accepted as potential contributors to the pathogenesis of IBS [3]. Accumulating evidence has demonstrated that alterations in gut microbiota and dysregulation of the brain-gut axis may be associated with IBS pathogenesis [4–7]. Moreover, the gut microbiota may be involved in IBS pathogenesis through the production of metabolites such as short-chain fatty acids (SCFAs) [8]. In the brain-gut axis, the neurotransmitter serotonin (5-hydroxytryptamine, 5-HT), associated with visceral hypersensitivity and gastrointestinal permeability, has attracted much attention [9, 10]. Mounting evidence detailing the bidirectional interactions between the gut microbiota and the brain supports the concept of the brain-gut-microbiota (BGM) axis, integrating the central nervous system (CNS), gastrointestinal tract (GIT), and microbiota [11, 12].

The brain-gut axis is the interactional two-way regulating axis of the CNS and gastrointestinal function which comprises the CNS, enteric nervous system (ENS), neuroendocrine and immune system [13]. 5-HT, as an important neurotransmitter in the brain-gut axis, may regulate gastrointestinal motility, visceral sensing, and mucosal secretion by activating both intrinsic excitatory and inhibitory enteric motor neurons. While cholinergic neurons can be stimulated by 5-HT to release acetylcholine and further contract smooth muscle, 5-HT can also irritate inhibitory nitrergic neurons to produce nitric oxide to relax smooth muscle [7]. An excess of 5-HT may trigger primary neuronal afferents, leading to visceral hyperalgesia [14]. Thus, in recent years, 5-HT has attracted considerable attention due to its potential effects on gastrointestinal motility, visceral sensory function, and psychophysiology in IBS [15].

Studies worldwide have provided convincing evidence that gut microbiota alterations may be associated with IBS [3, 4, 16]. However, the causal relationship remains unclear. Interactions between the gut microbiota and host cells on the gut mucosal surface could affect the intestinal microenvironment. Conversely, an altered intestinal microenvironment could modify the gut microbiota [17]. SCFAs are the main metabolites of the gut microbiota and may stabilize the intestinal microenvironment and modulate the gut microbiota [18]. Chassard et al. [19] reported dysbiosis of multiple microbes in IBS patients, and a reduction in *Rhodobacillus coli* may directly lead to SCFA deficiency, ultimately resulting in the visceral pain of IBS. In addition, with elevated concentrations of SCFAs, the pH of the intestinal tract could be reduced. At low pH, the proliferation of probiotic bacteria may be promoted while some pathogenic bacteria may be inhibited [20].

It has been reported that SCFAs and 5-HT might be involved in the bidirectional interactions of the BGM axis [21, 22]. Alterations in SCFAs and 5-HT in IBS patients have been demonstrated in some studies, and these alterations may influence gut motor activity and induce various physical symptoms [10, 23, 24]. However, there remains no consensus regarding the role of alterations in SCFAs and 5-HT in the pathogenesis of IBS. Therefore, we aimed to compare alterations in SCFAs and 5-HT in IBS patients and healthy controls (HCs) to explore their potential involvement in the BGM axis in IBS. We performed a meta-analysis to provide more definitive information on whether alterations in SCFAs and 5-HT between IBS patients and HCs contribute to IBS. Our findings provide insight for further explorations of the roles of SCFAs and 5-HT in the BGM axis and their participation in the pathogenesis of IBS.

## Methods

### Information sources and search strategy

In this meta-analysis, we searched the PubMed, Web of Science, Cochrane Library, and Scopus databases to identify relevant articles up to September 2018. We used “irritable bowel syndrome”, “IBS”, “short-chain fatty acids”, “SCFAs”, “serotonin”, “5-hydroxytryptamine”, and “5-HT” as the search terms. We further retrieved the references from included studies to search potential articles. Finally we selected articles according to the following inclusion and exclusion criteria.

### Inclusion and exclusion criteria

Studies were included if they were case–control studies detecting SCFAs and 5-HT in IBS patients and HCs. Below are the inclusion criteria to our meta analysis: (a) studies about IBS patients and HCs; (b) the participants are adults (both IBS patients and HCs); (c) studies measuring SCFAs or 5-HT; (d) available data in the articles and can eventually be expressed as mean ± SD; and (e) sufficient data to calculate the standardized mean difference (SMD) with 95% confidence interval (CI) between the IBS patients and HCs. Two researchers selected the studies that met the predetermined inclusion criteria independently. All potentially relevant papers were obtained and evaluated in detail. Any disagreement between researchers was resolved through discussion until consensus.

Below are the exclusion criteria to our meta analysis: (a) not a case–control study; (b) not adult participants; (c) no full text; and (d) no available or enough data. According to the inclusion and exclusion criteria, 10 articles (5 articles on SCFAs and 5 on 5-HT) were selected for our meta-analysis.

### Data extraction

We extracted the following data after reviewed every article: title of the article, name of the first author, year of publication, country, diagnostic criteria of IBS, age and sex of participants, number of IBS patients, number of HCs, methods by which levels of SCFAs and 5-HT were analyzed, available data expressed as mean ± SD (Tables 1, 2, 3, 4, 5), and study samples of the article. Disagreements regarding data extraction were resolved through discussion until consensus.

**Table 1:** The total SCFAs in IBS patient and HC

References	IBS patient			HC		
	Mean (mmol/l)	SD	Number	Mean (mmol/l)	SD	Number
Kopecny and Simunek [27]	88.15	207.44	16	108.95	60.01	5
Mortensen et al. [8]	96.89	3.64	18	114.00	19.00	9
Ringel-Kulka et al. [26]	92.10	44.10	114	92.00	33.30	33
Tana et al. [25]	107.10	31.10	26	85.80	28.60	26
Vernia et al. [28]	131.40	62.60	38	108.50	58.30	50

SCFAs, short-chain fatty acids; IBS, irritable bowel syndrome; HC, healthy control

**Table 2:** The acetic acid in IBS patient and HC

References	IBS patient			HC		
	Mean (mmol/l)	SD	Number	Mean (mmol/l)	SD	Number
Kopecny and Simunek [27]	60.16	126.08	16	73.26	44.70	5
Mortensen et al. [8]	62.67	3.14	18	73.00	11.00	9
Ringel-Kulka et al. [26]	58.00	25.80	114	56.60	18.50	33
Tana et al. [25]	67.00	18.80	26	56.70	18.00	26
Vernia et al. [28]	83.20	43.70	38	70.00	49.80	50

IBS, irritable bowel syndrome; HC, healthy control

**Table 3:** The propionic acid in IBS patient and HC

References	IBS patient			HC		
	Mean (mmol/l)	SD	Number	Mean (mmol/l)	SD	Number
Kopecny and Simunek [27]	18.06	45.00	16	26.08	13.53	5
Mortensen et al. [8]	18.11	2.05	18	21.00	5.00	9
Ringel-Kulka et al. [26]	18.20	9.80	114	20.00	8.00	33
Tana et al. [25]	20.50	9.20	26	15.30	6.60	26
Vernia et al. [28]	23.70	13.10	38	17.10	8.00	50

IBS, irritable bowel syndrome; HC, healthy control

**Table 4:** The butyric acid in IBS patient and HC

References	IBS patient			HC		
	Mean (mmol/l)	SD	Number	Mean (mmol/l)	SD	Number
Kopecny and Simunek [27]	9.93	23.92	16	9.16	4.94	5
Mortensen et al. [8]	10.00	1.67	18	12.00	3.00	9
Ringel-Kulka et al. [26]	15.80	12.10	114	15.40	8.90	33
Vernia et al. [25]	18.60	15.10	38	13.00	8.50	50

IBS, irritable bowel syndrome; HC, healthy control

**Table 5:** The 5-HT in IBS patient and HC

References	IBS patient			HC		
	Mean (nmol/l)	SD	Number	Mean (nmol/l)	SD	Number
Thijssen et al. [32]	10.99	22.75	154	10.84	13.44	137
Keszthelyi et al. [33]	26.20	18.20	15	1.90	1.36	15
Yu et al. [30]	35.31	2.15	30	10.55	1.35	30
Park et al. [29]	85.75	76.67	21	31.49	23.49	13
Dunlop et al. [31]	29.53	7.35	15	41.00	6.90	15

5-HT, 5-hydroxytryptamine; IBS, irritable bowel syndrome; HC, healthy control

### Quality assessment of included studies

The quality of each study was assessed by our study team according to the Newcastle–Ottawa Scale for case–control studies, which includes three aspects: selection, comparability, and exposure or outcome evaluation in the study population. The selection criteria comprise four items: (a) is the case definition adequate? (b) representativeness of the cases, (c) selection of the controls, and (d) determination of controls. The comparability criteria include comparability of cases and controls on the basis of the design or analysis. The exposure criteria comprise three items: (a) ascertainment of exposure, (b) same method of ascertainment for cases and controls, and (c) non-response rate. Disagreements were resolved through discussion until consensus.

### Statistical analysis

We performed a meta-analysis of alterations in SCFAs and 5-HT in IBS patients. REVIEW MANAGER 5.3, developed by staff at the Australasian Cochrane Centre in 2008 and revised in June 2014 to incorporate new features, was used for statistical analysis of continuous data. When the necessary continuous data were available, we calculated the SMD with 95% CI for the data. The I^2^ statistic was used to examine heterogeneity across studies. An I^2^ value close to 0% implies low heterogeneity, while an I^2^ value close to 100% implies high heterogeneity. In cases of obvious heterogeneity (I^2^ value > 50%), a random effects meta-analysis model was used.

### Funnel plot analysis for publication bias

A funnel plot was used to assess publication bias. Publication bias leads to asymmetry of the funnel plot, as the dispersion of smaller samples is larger and scatters more widely at the bottom of the graph, while the dispersion of larger sample is smaller, resulting in a narrower spread at the top of the plot.

## Results

### Study selection

**Fig. 1:**
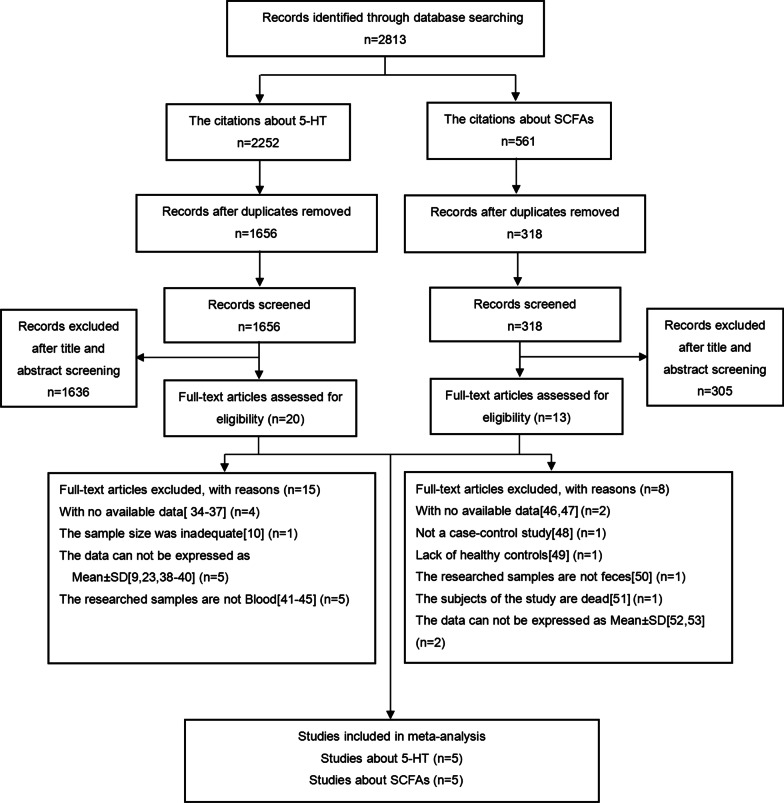
Flow diagram of assessment of studies identified in the meta-analysis

As shown in Fig. 1, a total of 2813 citations were obtained for review of titles and abstracts. After excluding duplicate and irrelevant articles, the remaining 13 articles on SCFAs and 20 articles on 5-HT was retrieved for review. Twenty-three studies were excluded for various reasons, as described in the flowchart (Fig. 1). Finally, five articles [8, 25–28] on SCFAs and five articles [29–33] on 5-HT were included in our meta-analysis. The two reviewers from our team were in full agreement in selecting these 10 studies.

### Study characteristics

The characteristics of the studies are summarized in Tables 6 and 7. A total of 212 IBS patients and 123 HCs were included in the 5 studies on SCFAs. In all studies, SCFAs were analyzed from fecal samples. Four studies measured SCFA levels by gas chromatography, while one used high-pressure liquid chromatography (HPLC). Data from four studies were expressed in the form of mean ± SD and from one study as mean ± SEM. We then converted the mean ± SEM into the mean ± SD. SCFA levels are expressed as concentrations in mmol L^−1^. We analyzed alterations in total SCFAs, acetic acid, propionic acid, and butyric acid in IBS patients.

**Table 6:** Characteristics of the five studies of SCFAs in the meta-analysis

References	Year	Location	n, IBS	n, Control	IBS diagnosis	Control composition	Age, IBS (range, ${\overline{\text{x}}} + {\text{s}}$)	Female IBS, n	Age, HC (range, ${\overline{\text{x}}} + {\text{s}}$)	Female HC, n	Sample	Technique
Ringel-Kulka et al. [26]	2015	America	114	33	Rome III	Healthy controls	35.4 ± 11.3	98	33.9 ± 13.0	29	Feces	Gas chromatography
Tana et al. [25]	2010	Japan	26	26	Rome II	Healthy controls	21.7 ± 2.0	13	21.9 ± 3.9	13	Feces	HPLC
Kopecny and Simunek [27]	2002	Czech	16	5	Not reported	Healthy controls	Not reported	Not reported	Not reported	Not reported	Feces	Gas chromatography
Vernia et al. [28]	1987	Italy	38	50	Not reported	Healthy controls	Not reported	Not reported	Not reported	Not reported	Feces	Gas liquid chromatograph
Mortensen et al. [8]	1987	Denmark	18	9	Not reported	Healthy controls	26–68	14	Not reported	Not reported	Feces	Gas liquid chromatography

SCFAs, short-chain fatty acids; IBS, irritable bowel syndrome; HC, healthy control; HPLC, high-pressure liquid chromatography

**Table 7:** Chracteristics of the included studies of 5-HT in this meta-analysis

References	Year	Location	n, IBS	n, Control	IBS diagnosis	Control Composition	Age, IBS (range, ${\overline{\text{x}}} + {\text{s}}$)	Female IBS, n	Age, HC (range, ${\overline{\text{x}}} + {\text{s}}$)	Female HC, n	Sample	Technique
Yu et al. [30]	2016	China	30	30	Not reported	Healthy controls	39 ± 21	14	42.5 ± 19.5	15	Serum	HPLC
Dunlop et al. [31]	2005	Britain	15	15	Rome II	Healthy controls	35.1 ± 2.9	15	35.9 ± 2.6	10	Plasma	HPLC
Thijssen et al. [32]	2015	Netherlands	154	137	Rome III	Healthy controls	44.5 ± 16.3	108	44.2 ± 19.3	84	Plasma	HPLC
Park et al. [29]	2009	Korea	21	13	Rome II	Healthy controls	24–78	13	29–68	6	Plasma	HPLC
Keszthelyi et al. [33]	2013	Netherlands	15	15	Rome III	Healthy controls	44 ± 13	8	33 ± 17	10	Plasma	HPLC

5-HT, 5-hydroxytryptamine; IBS, irritable bowel syndrome; HC, healthy control; HPLC, high-pressure liquid chromatography

For the five studies on 5-HT, 235 IBS patients and 210 HCs were included in our study. In all studies, 5-HT was analyzed in blood samples (four in plasma samples and one in serum sample) and measured by HPLC. The data from three studies were expressed as the mean ± SD, one study as the mean ± SEM, and one study as the median and range. We converted the available data to the form of mean ± SD where necessary. Concentrations of 5-HT are expressed as nmol L^−1^. Among the 10 articles included in our study, IBS patients and HCs were age- and sex-matched in 7 studies.

### Assessment of study quality

As shown in Table 8, we used Newcastle–Ottawa Scale to evaluate the quality of each case–control study. All studies included were of moderate quality but did not influence the eligibility of the studies.

**Table 8:** Quality assessment of the included studies

References	Year	Selection	Comparability	Exposure	Total
Ringel-Kulka et al. [26]	2015	4	1	2	7
Tana et al. [25]	2010	4	1	2	7
Kopecny and Simunek [27]	2002	2	1	2	5
Vernia et al. [28]	1987	2	1	2	5
Mortensen et al. [8]	1987	2	1	2	5
Yu et al. [30]	2016	2	1	2	5
Dunlop et al. [31]	2005	4	1	2	7
Thijssen et al. [32]	2015	4	1	2	7
Park et al. [29]	2009	4	1	2	7
Keszthelyi et al. [33]	2013	4	1	2	7

Selection includes four criteria: (1) is the determination of case adequate? (2) the representation of the cases, (3) the selection of the controls, (4) The determination of the controls; Comparability criteria include the Comparability of cases and controls is taken into account in design and statistical analysis; Exposure contains three criteria: (1) determination of exposure factors, (2) use the same method to determine the exposure factors in case and control group, (3) non-response rate

### Meta-analysis of SMD

**Fig. 2:**
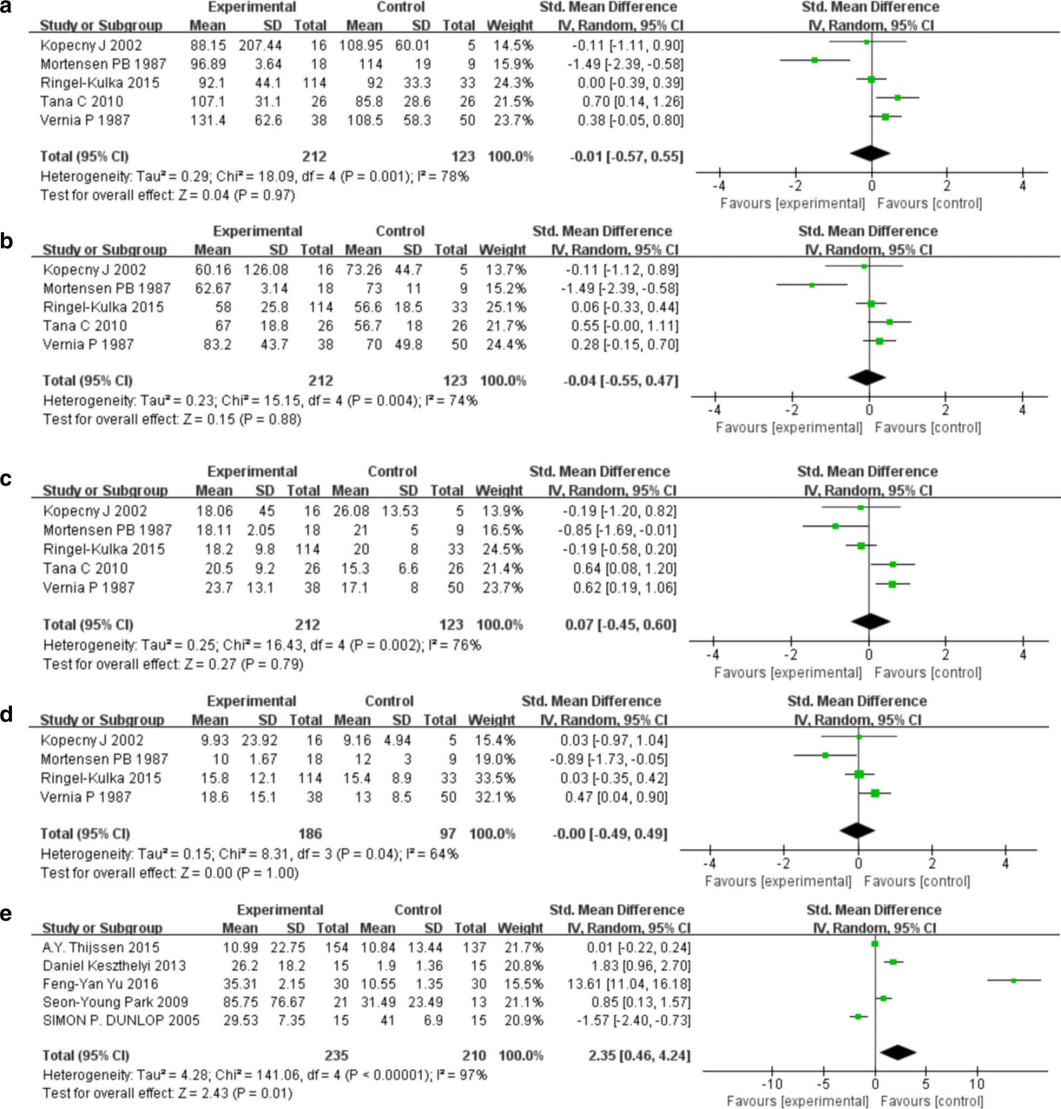
Forest plots of alterations of SCFAs and 5-HT in IBS patients versus HCs: **a** total SCFAs, **b** acetic acid, **c** propionic acid, **d** butyric acid, **e** 5-HT

Our meta-analysis analyzed alterations in SCFAs (total SCFAs, acetic acid, propionic acid, and butyric acid) and 5-HT in IBS patients from feces and blood respectively. As compared to HCs, the SMD of total SCFAs was − 0.01 (95% CI − 0.57–0.55), that of acetic acid was − 0.04 (95% CI − 0.55–0.47), that of propionic acid was 0.07 (95% CI − 0.45–0.60), and that of butyric acid was − 0.00 (95% CI − 0.49–0.49), as shown in Fig. 2a–d. There was significant heterogeneity in the studies included, with I^2^ values over 50%.

As shown in Fig. 2e, the SMD of 5-HT in IBS patients was 2.35 (95% CI 0.46–4.24). There was also significant heterogeneity in the studies included, with an I^2^ value of 97%.

### Analysis of funnel plots for publication bias

**Fig. 3:**
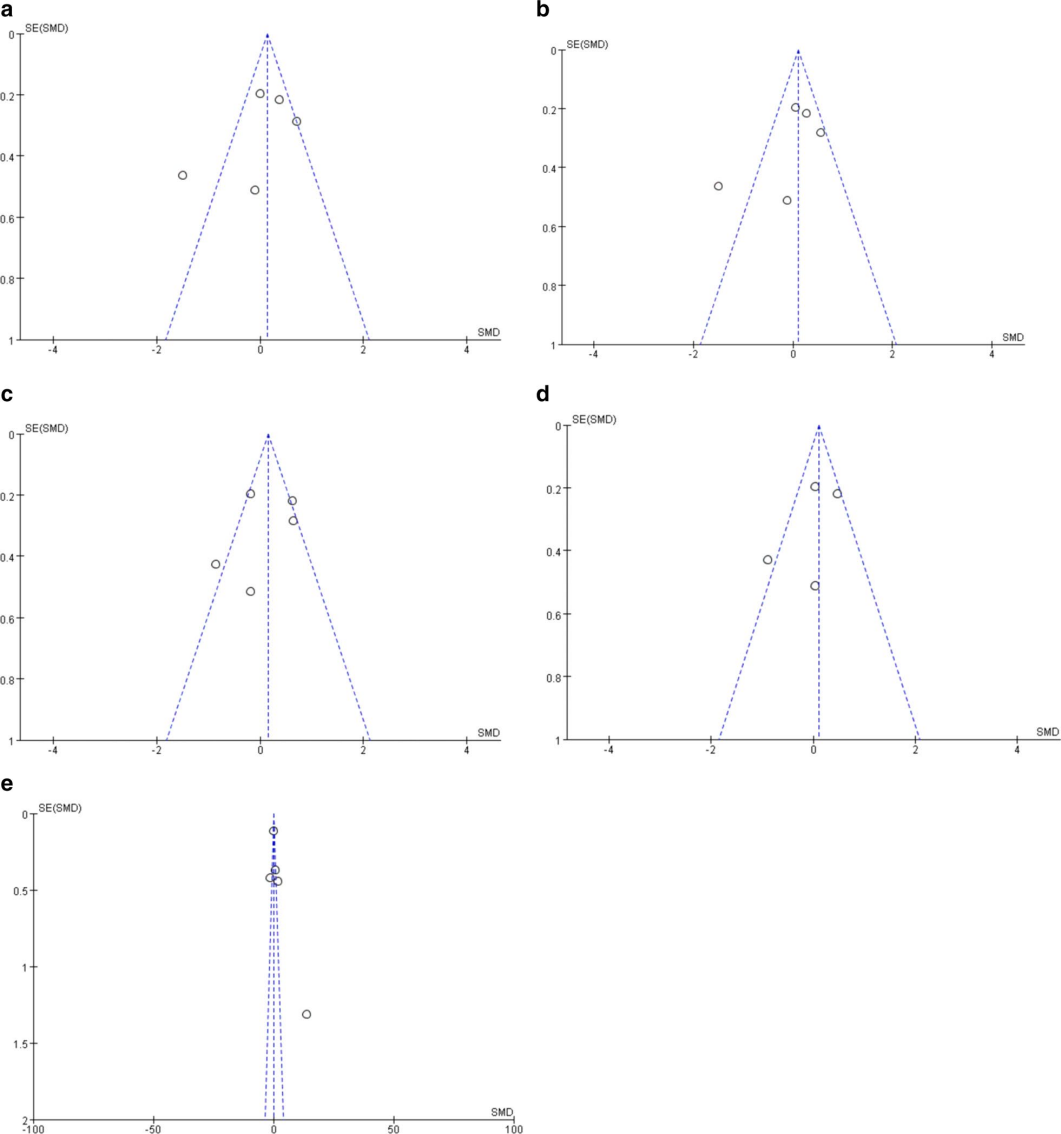
Funnel plots of alterations of SCFAs and 5-HT in IBS patients versus HCs: **a** total SCFAs, **b** acetic acid, **c** propionic acid, **d** butyric acid, **e** 5-HT

As shown in Fig. 3a–e, funnel plots were used to assess publication bias among the studies included. However, as the number of included studies was below 10, the ability of these funnel plots to detect publication bias is limited.

## Discussion

In recent years, alterations in bidirectional brain-gut interactions have been considered to play a vital role in IBS and some related functional gastrointestinal diseases [18, 54]. Alterations in the gut microbiota have also been confirmed as mechanisms of IBS pathogenesis worldwide [3]. It has been generally accepted that a stable gut microbiota is essential for normal gut physiology and contributes to appropriate signaling along the brain-gut axis [55]. SCFAs, as the main metabolites of the gut microbiota, and 5-HT, as an important regulatory factor of the brain-gut axis, may be involved in the abnormal BGM axis of IBS. Alterations in SCFAs in feces and 5-HT in blood have been found in IBS patients in some studies [27, 28, 30–32]. This is the first meta-analysis to explore the alterations in SCFAs and 5-HT in IBS, and some interesting findings were observed in our meta-analysis. We found that 5-HT levels in the blood in IBS patients were increased compared to those in HCs. However, we found no significant differences in SCFAs in feces between IBS patients and HCs.

Ninety-five percent of human 5-HT comes from the intestines, mainly the intestinal EC cells of the mucous layer, which contains tryptophan hydroxylase (TPH), necessary for the synthesis of 5-HT. The release of 5-HT in EC cells is sensitive to the pressure and chemical stimulation in the lumen [56]. In our study, we found that 5-HT levels were increased in the blood in IBS patients compared to those in HCs. Only one study by Dunlop et al. [31] reported lower 5-HT levels in IBS patients. In the 5 studies on 5-HT in our meta-analysis, all researchers used high-pressure liquid chromatography (HPLC) to measure 5-HT levels in subjects. However, their findings were inconsistent, which may imply that the HPLC method is unstable and has poor repeatability. We did not analyze 5-HT levels among different subtypes of IBS patients in our study. However, some studies have found that differential alterations in 5-HT in various IBS subtypes. Atkinson et al. [9] reported that 5-HT levels were reduced in patients with constipation-predominant IBS (IBS-C). Bearcroft et al. [10] and Houghton et al. [24] found increased 5-HT levels in patients with diarrhea-predominant IBS (IBS-D). These inconsistent findings indicate that alterations in 5-HT may be related to the symptoms of constipation or diarrhea in IBS patients. Houghton et al. [24] also reported that food intake could stimulate colonic motor activity, alter visceral sensitivity, increase blood 5-HT levels, and lead to abdominal pain or diarrhea in IBS patients. Hence, we speculate that alterations in 5-HT may be related to IBS pathogenesis. Our findings support the idea that 5-HT may be a therapeutic target for IBS. In fact, some drugs that act on 5-HT signaling are currently used to treat IBS. Tegaserod, a 5-HT4 partial agonist and alosetron, a 5-HT3 antagonist is used in IBS-C and IBS-D to relieve symptoms, respectively. Other agents regulating the levels of 5-HT, such as tricyclic antidepressants and serotonin selective reuptake inhibitors, have also been used in some patients with IBS [56]. Although we found differences in 5-HT levels in blood between IBS patients and HCs, further studies to explore the correlation between altered 5-HT levels and IBS symptoms are warranted.

SCFAs are a type of saturated fatty acids which including acetic acid, propionic acid, butyric acid, isobutyric acid, isopentoic acid, caproic acid, and isocaproic acid. The main SCFAs distributed in the intestines are acetic acid, propionic acid, and butyric acid. Most SCFAs are absorbed by the intestines, while only low levels of SCFAs escape absorption and can be detected in fecal samples [57]. In our study, we found there were no significant differences in total SCFAs in the feces between the IBS patients and HCs from the available data. We then explored differences in acetic acid, propionic acid, and butyric acid between IBS patients and HCs. Similarly, there were no significant differences in acetic acid, propionic acid, or butyric acid between IBS patients and HCs. Although there were no significant alterations in SCFAs in IBS patients, some studies have reported that SCFAs are altered in the feces of IBS patients [25, 27, 28]. Tana et al. [25] found that levels of acetic acid, propionic acid, and total SCFAs were significantly higher in IBS patients than in controls. These findings were similar to the results reported by Vernia et al. [28]. However, Kopecny et al. [27] found that the fecal SCFAs of IBS patients were characterized by lower levels of total SCFAs, acetic acid, and propionic acid and by higher levels of butyric acid. Although there is no consensus regarding alterations in SCFAs in IBS patients, the altered SCFAs reported in the abovementioned studies may imply their involvement in the pathogenesis of IBS. Interestingly, some studies have reported that SCFAs are associated with symptoms in IBS patients. Soret et al. [58] found that SCFAs could induce plasticity of intestinal myometrial neurons and promote colonic motility, which may cause diarrhea in IBS patients. In addition, Cherbut et al. [59] and Grider et al. [60] reported that the activity of SCFAs in regulating colonic motility may be affected by their concentrations. Thus, a low concentration (10–100 mmol L^−1^) of SCFAs could increase colonic motility or have no effect, while a high concentration (> 100 mmol L^−1^) of SCFAs could inhibit colonic motility. Maintenance of gut microbiota homeostasis could release SCFAs to maintain intestinal mucosal permeability [61]. Dysbiosis of the gut microbiota may lead to insufficient SCFA intake in IBS patients, which directly constrains the distribution of compact connexin and then increases intestinal mucosal permeability [62]. Regulating SCFAs through the intestinal microenvironment may be a potential therapeutic target for IBS. Clinical trials have found that relevant SCFA products can improve IBS symptoms; for example, sodium butyrate reduced visceral pain in IBS patients [53, 63]. Although some studies have reported alterations in SCFAs in feces of IBS patients and the association of these alterations with symptoms in IBS patients, we found no significant differences in SCFA levels in feces in IBS patients and HCs in our meta-analysis. This implies that the gut microbiota may be involved in the pathogenesis of IBS through pathways other than SCFAs, such as alterations in immune profiles, effects on the CNS, or modulation of the gut neuromuscular function [62], or most SCFAs are absorbed in the colon and the analyzed results from feces are not representative for SCFAs in other parts of the gastrointestinal tract. However, the lack of significant differences in SCFAs between IBS patients and HCs in our meta-analysis may also be due to inadequate sample sizes in the original studies. The analytical methods or samples used to measure SCFA levels also need improvement and optimization in future studies.

As our study indicated that the 5-HT was increased in blood with IBS patients. 5-HT is the significant signaling molecule in the BGM axis and Clarke et al. [64] found the gut microbiota regulated the synthesis of 5-HT in CNS, which might further suggest that the disorder of the BGM axis may be the pathogenesis of IBS.

There are some limitations to our study. First, the statistical heterogeneity was significant among the included studies. This could be explained by differences in analytical methods, sample size, and diagnostic criteria for IBS in the included studies. Second, some studies used intestinal mucosal tissues as samples to analyze 5-HT levels in IBS patients and HCs [33, 40–45]. Data from these studies could not be processed effectively and were not included in our meta-analysis. This may lead to a bias in our meta-analysis results. Lastly, there are four subtypes of IBS, including IBS-C and IBS-D. However, our meta-analysis did not analyze the various subtypes of IBS owing to the limited number of samples but just analyze the IBS-D, the validity of the results could be questioned. More complete profiles of alterations in fecal, blood, or mucosal metabolites in various IBS subtypes are need for a better understanding of their roles in the BGM axis of IBS.

## Conclusions

In conclusion, we found increased levels of 5-HT in IBS patients in our meta-analysis. However, there was no significant alteration of SCFAs between IBS patients and HCs. Our findings imply that alterations in 5-HT may be associated with the pathogenesis of IBS and affect the symptoms of IBS patients. Although we found no significant differences in SCFAs between IBS patients and HCs in our meta-analysis, some studies have reported that SCFAs are altered in IBS; such alterations in SCFAs may be a potential mechanism for the development or maintenance of symptoms in IBS patients. The findings of our study warrant further exploration of the relationship between the BGM axis and IBS. Therefore, further studies involving advanced molecular techniques and more strict experimental design are needed to explore alterations in SCFAs in IBS patients, which study samples would not just confined to feces.

## Data Availability

All data and materials during this study are presented within the manuscript.

## Abbreviations

SCFAs: Short-chain fatty acids
5-HT: 5-Hydroxytryptamine
IBS: Irritable bowel syndrome
BGM: Brain-gut-microbiota
SMDs: Standardized mean differences
CIs: Confidence intervals
HCs: Healthy controls
CNS: Central nervous system
GIT: Gastrointestinal tract
ENS: Enteric nervous system
SMD: Standardized mean difference
CI: Confidence interval
TPH: Tryptophan hydroxylase
IBS-C: Patients with constipation-predominant IBS
IBS-D: Patients with diarrhea-predominant IBS
